# Assessment of patients with a Chiari malformation type I

**DOI:** 10.1016/j.bas.2021.100850

**Published:** 2021-12-03

**Authors:** Sharon Ka Po Tam, Jonathan Chia, Andrew Brodbelt, Mansoor Foroughi

**Affiliations:** a Royal Sussex County Hospital, Brighton and Sussex University Hospitals NHS Trust, UK; b The Walton Centre, NHS Foundation Trust, UK

**Keywords:** Chiari, Syringomyelia, Basilar invagination, Tethered cord

## Abstract

**Introduction:**

The prevalence of Chiari malformation type I (CM-I) has been estimated as up to 1% of the general population. The majority of patients are asymptomatic and usually do not need treatment. Symptomatic patients, and some asymptomatic patients with associated conditions, may benefit from further assessment and treatment.

**Research question:**

The aim of this review was to describe the clinical and radiological assessment of patients presenting with a CM-I.

**Material and methods:**

A literature search was performed using the PubMed and Embase databases focused on clinical assessment and imaging techniques used to diagnose CM-I.

**Results:**

Following a complete clinical evaluation in patients with symptomatic CM-I and/or radiologically significant CM-I (tonsillar impaction, resulting tonsillar asymmetry and loss of CSF spaces), MRI of the brain and whole spine enables an assessment of the CM-I and potential associated or causative conditions. These include hydrocephalus, syringomyelia, spinal dysraphism, and tethered cord. Flow and Cine MRI can provide information on CSF dynamics at the craniocervical junction, and help in surgical decision-making. Hypermobility or instability at the upper cervical and craniocervical junction is less common and can be measured with CT imaging and flexion/extension or upright MRI.

**Discussion and conclusion:**

The majority of CM-I detected are incidental findings on MRI imaging of brain or spine, and do not require intervention. Once a radiological diagnosis and concern has been raised, clinical assessment by an appropriate specialist is required. A MRI brain and cervical spine is indicated in all radiologically labelled CM-I. In symptomatic patients or cases of radiologically significant CM-I, MRI of the brain and entire spine is indicated. Further investigations should be tailored to individuals’ needs.

## Highlights

• A CM-I assessment should include a history and examination and a referral to a specialist.
• MRI of the brain and spine enables an assessment of the CM-I and potential associated conditions.
• Flow and Cine MRI can help in surgical decision-making.

## 1 Introduction

Originally described by the Austrian pathologist Hans Chiari in 1891, Chiari malformations (CMs) are hindbrain malformations that vary in severity (Chiari, 1895). According to the Chiari classification system, type I – III CM are graded based upon an increasing degree of hindbrain herniation through the foramen magnum. Type IV CM represents cerebellar aplasia or hypoplasia (Hadley, 2002). Recently type 0 and 1.5 CM have also been proposed, with type 0 CM describing a patient with symptoms and a syrinx, with ‘0 ​mm’ of tonsilar descent, and type 1.5 referring to tonsillar herniation with additional caudal descent of the brainstem (Mariwalla et al., 2014). The term Complex Chiari has been used to describe cerebellar tonsilar herniation with another radiographic feature, such as a syrinx, medullary kink, cranial skull base abnormality, caudal descent of the brainstem, basilar invagination, or scoliosis (Brockmeyer, 2011).

Traditionally, the defining feature of the Chiari malformation type I (CM-I) is tonsillar descent of 5 ​mm or more beyond the foramen magnum (Elster and Chen, 1992). This definition is being questioned, as a more encompassing definition of symptomatic syndromes due to cerebrospinal fluid (CSF) obstruction, or compression at the craniocervical juction appears preferable, but no agreement has been reached, and the 5 ​mm rule is still used by most authors. Neuroradiology is essential in the diagnosis of CM-I to assess the anatomical structures and fluid dynamics that are associated with a CM-I. Magnetic resonance imaging (MRI), including dynamic and upright views, are described, as well as myelography and computed tomography (CT).

With the increasing use of neuroimaging, the number of ‘victims of modern imaging technology' is on the rise (Hayward, 2003). More than 1% of the population are being diagnosed with CM-I, yet the vast majority of CM-I detected are incidental, and do not require treatment (Meadows et al., 2000; Langridge et al., 2017). A new diagnosis of a Chiari malformation can produce anxiety in patients, and be used to explain a myriad of symptoms, many of which are not improved with surgical ‘correction’. The degree of tonsillar herniation alone does not correlate to its clinical significance, nor measure a functional deficit. The authors provide a review of the assessment for patients with a CM-I, including the newer radiological techniques that are increasingly being used.

### 1.1 Methods

A literature search was performed by two independent reviewers using PubMed and Embase databases focused on the clinical and radiological assessment used to diagnose CM-I. The following combined search terms: ‘Chiari AND clinical’, ‘Chiari AND MRI’, Chiari AND CT′, ‘Chiari AND syringomyelia’, ‘Chiari AND basilar invagination’ and ‘Chiari AND tethered cord’ were used. To further identify all potentially relevant studies, we manually searched reference lists from all the retrieved articles. No time limit was set during the search. Indexes were last accessed on October 26, 2021.

### 1.2 Study criteria

The inclusion criteria were as follows: articles that addressed clinical presentation and investigation for CM-I, and articles that evaluated CM-I associated conditions. All publications were limited to human subjects and written in English.

## 2 Results

### 2.1 Search results

The search strategy identified 2854 articles. After removing duplicate studies, our inclusion and exclusion criteria were applied to the titles of the remaining articles, yielding 64 articles.

Ten studies described clinical presentations and physical examination findings in CM-I patients and associated conditions. Three articles evaluated the use of plain films and computed tomography as the screening tool for scoliosis and initial diagnostic tool for CM-I respectively. Twenty-six articles demonstrated the use of magnetic resonance imaging of the brain, which described the correlation between tonsillar herniation, tonsillar configuration, size of posterior cranial fossa, hydrocephalus and CM-I. Twenty-three articles discussed the use of dynamic flow and motility studies to assess CM-I symptoms, predict post-operative outcomes, as well as hypermobility syndromes. Nine articles evaluated the use of MRI of the spine to assess syringomyelia and tethered cord syndrome.

### 2.2 Discussion

#### 2.2.1 Clinical assessment

**Fig. 1:**
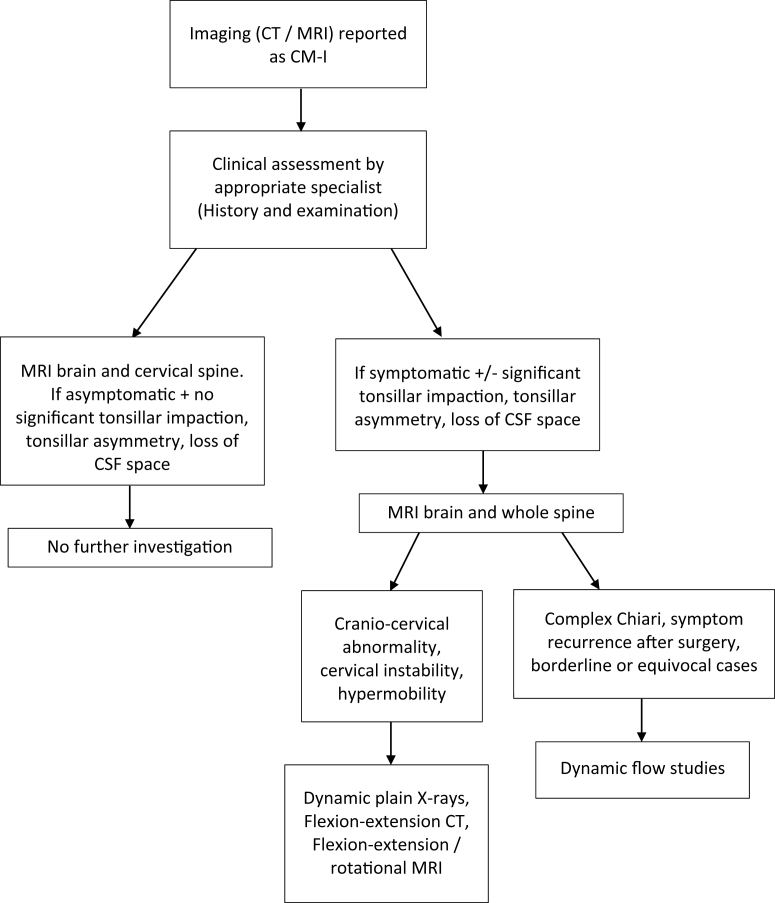
Diagnostic flowchart of Chiari malformation.

The first part of any assessment is to take a clinical history and perform a complete physical and neurological examination (Fig. 1). The examination should include an assessment of hypermobility (Malfait et al., 2017). The classic symptom of CM-I is a severe transient suboccipital headache, which is commonly aggravated by head dependency, postural changes, exertion, and the Valsalva maneuver (Mea et al., 2011). The pain may be caused by stretch of pain receptors at the foramen magnum, or a transient increase in pressure, although the true mechanism is not known. Chronic daily headache is common in CM-I patients, as in the general population (Taylor and Larkins, 2002). Otoneurological symptoms such as vertigo, nystagmus and tinnitus have been suggested to be due to cranial nerve traction from hindbrain descent (Mea et al., 2011). Bulbar and other brain stem dysfunction, such as vocal cord paralysis, hoarseness, palatal weakness, tongue atrophy, cricopharyngeal achalasia, sleep apnoea and nystagmus, are rare in CM-I patients, but more common in patients with associated skull base anomalies, and may be due to compression of the lower cranial nerves and medulla (Dyste et al., 1989; Li et al., 2005). Peripheral neurological examination may find signs of an associated syrinx (Cahan and Bentson, 1982).

Syringomyelia can cause a progressive dissociated sensory loss, with spinothalamic pain and temperature pathways affected more than the dorsal column's light touch and proprioception, which spread in a cape-like distribution (Honan and Williams, 1993; Grant et al., 1987). Other symptoms and signs include weakness, initially in the intrinsic hand muscles, burning dysesthesia, autonomic dysfunction, spasticity, and scoliosis. Autonomic dysautomnia, chronic tiredness, and postural tachycardia syndrome have all been ascribed to excessive stretching or compression of the brain stem in patients with hypermobility at the craniocervical junction, although evidence supporting this view remains controversial (Henderson et al., 2017). Bladder and bowel dysfunction, back and leg pain, and leg weakness can be an indication of a tethered cord. Finally, an external examination for dysraphic signs including midline posterior dimples or hair tufts should be performed. After the history and examination, an assessment is made of the likelihood of the symptoms being related to the CM-I and/or associated conditions, their severity, and the potential need for, and benefit from, treatment.

The clinical and radiological assessment should take into consideration the underlying cause, as investigation and treatment should be directed at the cause rather than the resultant CM-I. An assessment of more than 700 patients with CM-I showed a variety of causes, and provides some guiding principles (Milhorat et al., 2010a). The commonest was a small posterior fossa (less than 190 ​mL). Other causes included increased pressure from above due to hydrocephalus, idiopathic intracranial hypertension, or a mass lesion, or something pulling from below, such as a tethered cord or reduced spinal subarachnoid pressure due to a lumbar peritoneal shunt or chronic CSF leak. The final associated cause was a base of skull and upper cervical spine abnormalities that could be structural or due to hypermobility.

### 2.3 Imaging evaluation

#### 2.3.1 Plain films

Plain films are not generally used in the work up of patients with a CM-I, other than those with scoliosis. In children, up to 30% with a symptomatic Chiari and syrinx will develop scoliosis. The scholiosis in more than 50% of these children may be improved with treatment of their CM-I (Hwang et al., 2012). Plain X-rays of the entire spine are still used to assess, measure, and monitor the curve (Oestreich et al., 1998).

#### 2.3.2 Computed tomography (CT)

With the ready availability and widespread use of advanced neuroimaging modalities, many patients are still initially diagnosed based on a CT scan. Whilst most patients will proceed to MRI, CT remains essential in assessing the bony anatomy in patients with congenital or acquired bony abnormalities at the craniocervical junction, and may help with dynamic examination (see dynamic mobility studies below). Basiler invagination, platybasia, klippel feil, atlanto occipital assimilation, and other more complex anomalies may be seen (Elster and Chen, 1992). CT may also be used in CT myelography for the assessment of occult spinal CSF leaks. Whilst MR myelography may be more accurate concerns remain about the use of intrathecal gadolinium (Chazen et al., 2014).

### 2.4 Magnetic resonance imaging (MRI) - brain

#### 2.4.1 Tonsillar herniation

MRI of the brain and cervical spine remains the imaging modality of choice for the initial evaluation of CM-I. Traditionally, a radiological diagnosis of CM-I is made on the T1 or T2 sagittal midline view by measuring the perpendicular distance between the tip of the herniated tonsil and the foramen magnum (McRae's line). However, the tonsils exist in three dimensions, and can be unequal in size, shape, and projection. As such, coronal cuts may provide additional valuable information relating both to diagnosis and helping plan the surgical approach (Spinos et al., 1985).

Grading systems have been proposed, based on the degree of tonsillar herniation and the age of the patient. One group of authors suggested that the tonsils should be considered normal up to 3 ​mm, borderline between 3 and 5 ​mm, and pathologic when they exceed 5 ​mm (Aboulezz et al., 1985). Two further reports suggested age dependant cut off values of 6 ​mm up to 10 years, 5 ​mm 10–30 years, 4 ​mm 30–70 years, and 3 ​mm over 70 years (Smith et al., 2013; Mikulis et al., 1992). The ascent of the cerebellar tonsils with advancing age may have more to do with the gradual reduction in cerebral volume over time, rather than anything inherent in the CM-I itself.

As an absolute value, the number of mm of tonsillar descent is relatively unhelpful (Jussila et al., 2021), unless it is progressive which suggests increasing intracranial pressure or an ongoing pull from chronic spinal subarachnoid hypotension. Chiari 0 and Chiari 0.5 have been described, with patients with 0 ​mm or <5 ​mm respectively of tonsillar descent, with appropriate symptoms and improvement after surgery. The most important aspect of any radiological imaging is a clear assessment of the three-dimensional anatomy and CSF dynamics at the craniocervical junction.

### 2.5 Tonsillar configuration

**Fig. 2:**
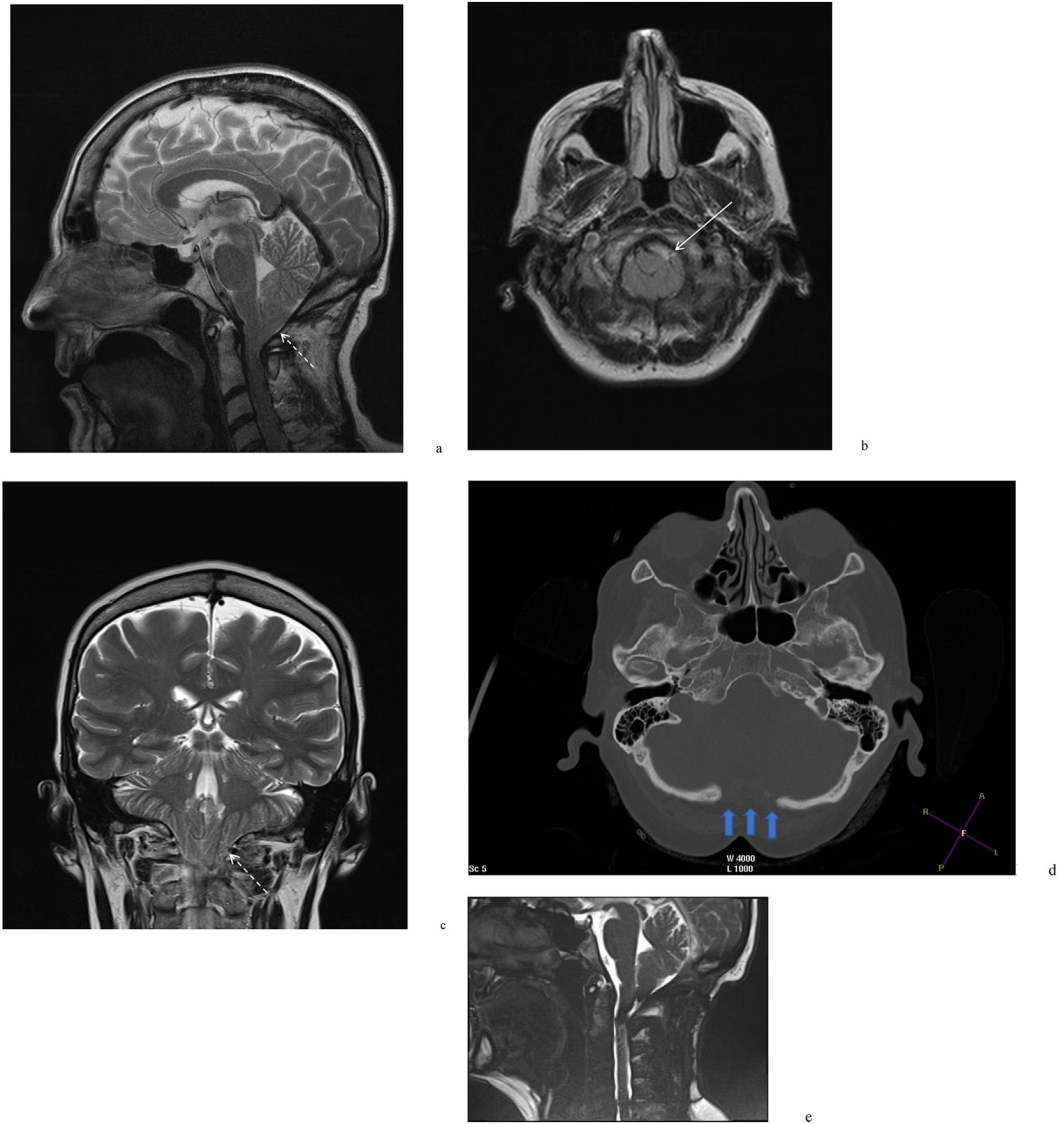
Sagittal (a), axial (b) and coronal (c) T2 weighted images from a brain MRI demonstrating crowding of the CSF spaces at the level of the foramen magnum (solid white arrow) and a peg-like deformity of the cerebellar tonsils (white dashed arrow). A decompressive posterior fossa craniectomy (blue arrows) was performed (d) with CSF flow voids along the craniocervical junction anteriorly and posteriorly which appear as areas of darker signal (e). (For interpretation of the references to colour in this figure legend, the reader is referred to the Web version of this article.)

The degree of tonsillar herniation has a poor correlation with the severity of symptoms, as up to 30% of patients with significant tonsillar herniation are asymptomatic (Elster and Chen, 1992). Instead, tonsillar shape has been used (Spinos et al., 1985; Meadows et al., 2000). Severe compression produces peg-like tonsils, which may further restrict CSF flow. Pegged tonsils are more common in patients with herniation >5 ​mm (85%) compared to rounded or intermediate shapes (Smith et al., 2013) (Fig. 2).

### 2.6 The size of posterior cranial fossa (PCF)

**Fig. 3:**
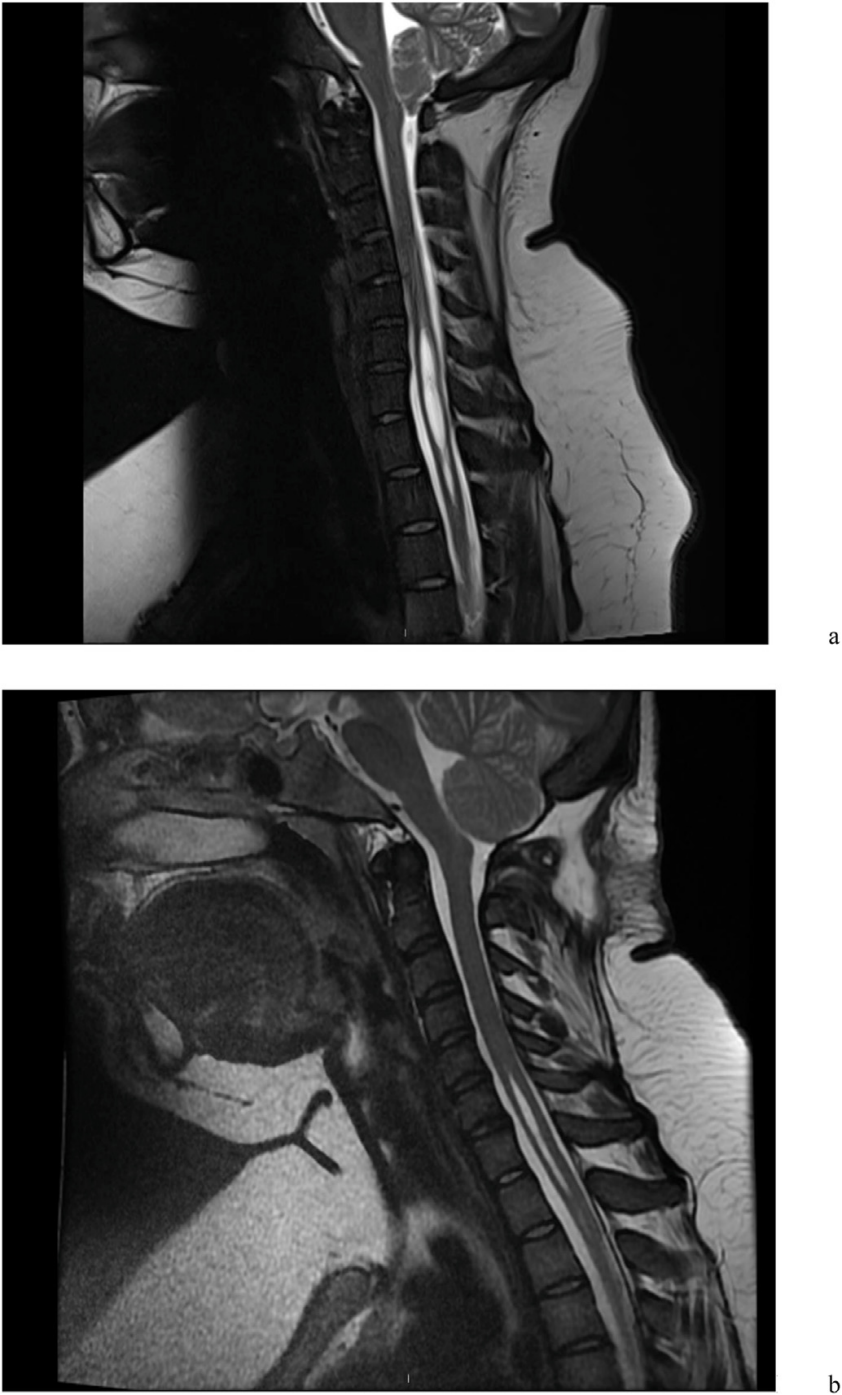
Different causes of tonsillar descent. I. Small posterior fossa. Sagittal T2 weighted C-spine MRI of a patient pre (a) and post (b) posterior fossa decompression with a patch due, showing the early reduction in syrinx size following enlargement of the posterior fossa, and tonsillar cautery.

The commonest cause of a CM-I is thought to be an abnormality of the paraxial mesoderm, resulting in a developmental mismatch between the neural and bony structures (Milhorat et al., 1999). The size of the PCF can be measured using linear markers. The lengths of the clivus, supraocciput, and exocciput are often shorter in patients with CM-I compared with healthy individuals, although the normal measurements can vary significantly (Milhorat et al., 1999; Nishikawa et al., 1997; Karagöz et al., 2002). One group proposed that occipital bone hypoplasia and reduced PCF volume, with no aetiological co-factors, were called classical CM-I (Milhorat et al., 2010a) (Fig. 3). Using measurements of the osseous PCF area, the clival length, the distance between the corpus callosum, pons, and the FM, a second group produced a probability model to predict CM-I symptomatology regardless of the degree of tonsillar herniation with 93% sensitivity and 92% specificity (Urbizu et al., 2014). Similar morphometric findings were observed in patients with Chiari-like symptomatology without significant cerebellar tonsillar herniation (Sekula et al., 2005; Dufton et al., 2011). However, others have found no association between the size of PCF and clinical symptomatology (Stovner et al., 1993). Due to the lack of agreement between studies, at present, linear measurements of the posterior fossa do not appear to provide any additional information in radiological assessment.

There has been interest in investigating volumetric approaches. The posterior fossa volume is about 190 ​mL in normal individuals (Milhorat et al., 2010b). A volume ratio (brain volume divided by cranial volume in the PCF) to measure crowding was investigated and was significantly larger in CM-I when compared to that of healthy individuals (Nishikawa et al., 1997). Furthermore, a smaller ratio of PCF to supratentorial volume correlated to a better post-operative clinical outcome, as did the extent of the craniectomy and the degree of PCF volume increase (Badie et al., 1995). The optimal PCF volume increase and extent of craniectomy required could then be predicted on the basis of the pre-operative MRI (Noudel et al., 2011). Despite these potential diagnostic and predictive benefits, PCF volume assessments are rarely used in clinical practice due to the laborious process of volume calculation, but may become useful as automatic segmentation algorithms improve.

### 2.7 Hydrocephalus

Chiari believed that CM were secondary to long standing hydrocephalus, although hydrocephalus and idiopathic intracranial hypertension (IIH) are seen in only 7–11% of patients with CM-I (Milhorat et al., 1999; Tubbs et al., 2003). A causal relationship may be different in different patients. It may be that occlusion of the foramen of Magendie and an associated CM-I obstruct IVth ventricle outflow leading to hydrocephalus, or that hydrocephalus or IIH leads to the downward herniation of the tonsils causing a CM-I (Orakdogen et al., 2015; Decq et al., 2001). As part of the initial clinical assessment of the patient, symptoms and signs of hydrocephalus are examined for, and if found, treatment should be directed at the hydrocephalus rather than the CM-I in the first instance (Hayhurst et al., 2008). MRI brain, MR or CT venography, intracranial pressure monitoring, and venous pressure measurements can help establish treatment options.

### 2.8 Magnetic resonance imaging (MRI) – craniocervical junction – dynamic evaluation

#### 2.8.1 Dynamic flow studies

Dynamic studies have been used to assess CM-I symptoms and predict post-operative outcomes. In normal individuals, CSF at the craniocervical junction flows in a pulsatile cranial, then caudal motion synchronized with cardiac and respiratory induced changes in intracranial blood volume (Wilson, 2016). The spinal arachnoid space acts as a buffer to limit intracranial pressure peaks (Whedon and Glassey, 2009). In children, greater intracranial compliance results in the faster caudal velocity (Ohara et al., 1988). Phase contrast cine MRI in symptomatic CM-I patients has identified greater fluctuations of CSF velocity in different regions, with a higher peak velocity at the FM (4.8 ​cm/s vs 3.3 ​cm/s in healthy controls), and a reduction in overall volume movement (Haughton et al., 2003; Quigley et al., 2004; Armonda et al., 1994). Flow jets are described, with regions with a preponderance of flow in one direction, and synchronous bidirectional flow (Quigley et al., 2004). Abnormal pulsatile motion of the cerebellar tonsils are observed in symptomatic CM-I patients, and the amplitude of tonsillar pulsation and the degree of arachnoid space reduction improves following surgery. (Wolpert et al., 1994; Pujol et al., 1995).

**Fig. 4:**
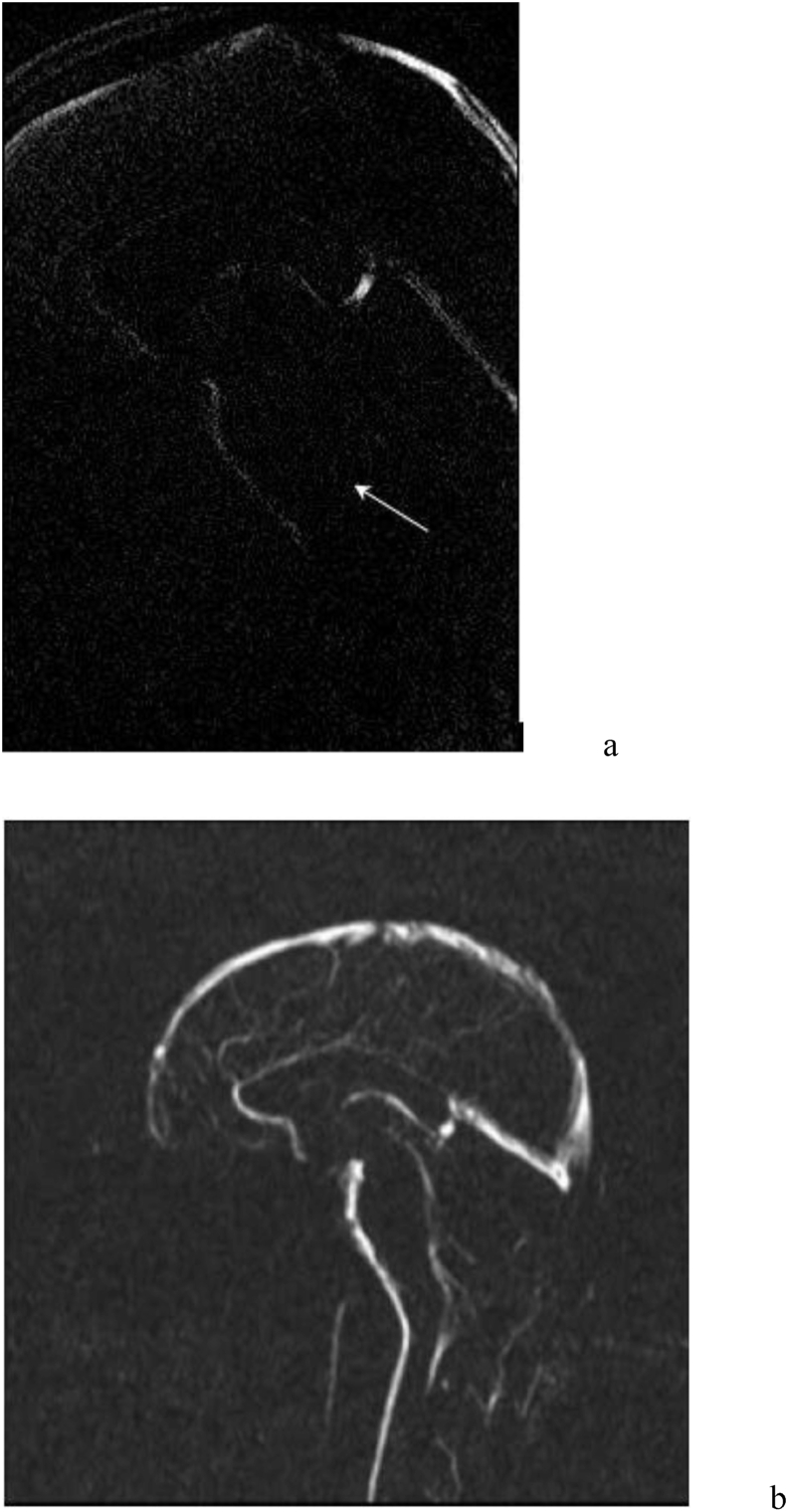
Selected image from an MRI of the brain including velocity encoded phase contrast flow study sequence (a). This reveals tonsillar descent of 17 ​mm. The absence of CSF flow posterior to the cervico-medullary junction is marked by the arrow (a). A normal appearing CSF flow sequence of comparison (b) (Battal et al., 2011).

CSF dynamic studies have been used to distinguish symptomatic from asymptomatic CM-I patients, yet the evidence remains inconsistent (Hofkes et al., 2007; Krueger et al., 2010). However, CSF velocity patterns in CM-I patients may be useful in predicting surgical improvement (Bhadelia et al., 1995; McGirt et al., 2006; Ventureyra et al., 2003; Armonda et al., 1994). A group of patients with normal preoperative hindbrain CSF flow were 4.8-fold more likely to experience symptom recurrence post-operatively, irrespective of their degree of tonsillar herniation or presence of syringomyelia, whilst complete CSF flow obstruction before surgery was associated with the long-term resolution of symptoms (McGirt et al., 2006). At present, dynamic studies provide an assessment of the degree of obstruction at the craniocervical junction, and may help predict surgical suitability. They are not useful in every case, but obstructed flow can be used to help predict a more successful surgical outcome in borderline cases, or for follow up assessment in symptom recurrence (Fig. 4).

### 2.9 Dynamic mobility studies

**Fig. 5:**
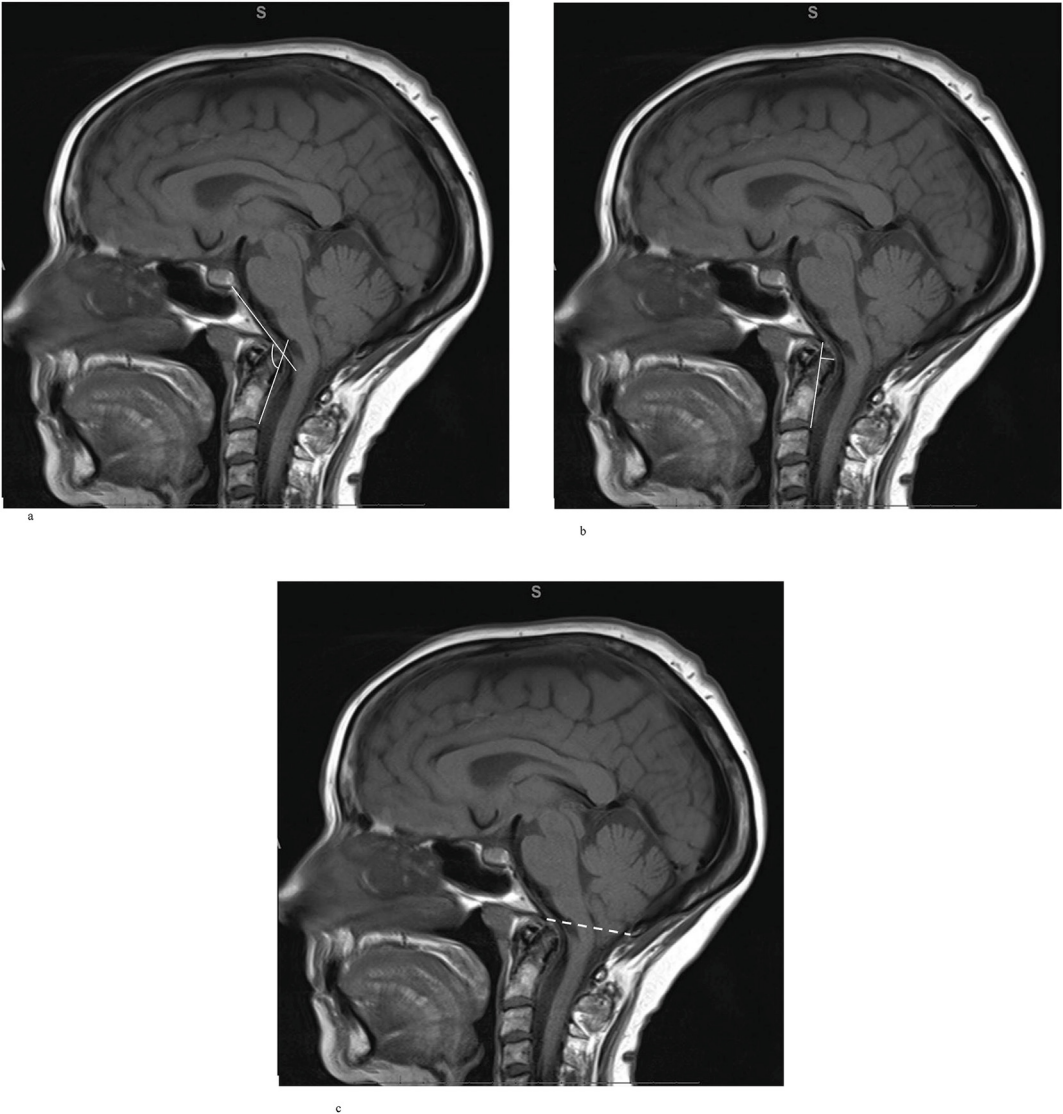
(a) The Clivoaxial angle (CXA) measures flexion at the craniocervical junction by measuring the angle between a line drawn along the clivus with that along the posterior margin of the odontoid peg and is said to be abnormal if on a flexion C-spine MRI it is < 135°. (b) The pBC2 line examines the dorsal protrusion of the odontoid peg, and is said to be abnormal if ≥ 9 mm. (c) McRae's line is the line drawn across the inferior margin of the foramen magnum, and is used to measure tonsilar descent.

CM-I has been linked to hypermobility syndromes, such as Ehlers-Danlos, and Marfans syndrome. (Henderson et al., 2017; Milhorat et al., 2010a). Hypermobility syndromes are a heterogenous group of inherited connective tissue disorders characterized by joint hypermobility, skin extensibility, and tissue fragility (Forghani, 2019). It has been suggested that hypermobility or instability at the craniocervical junction causes basiler invagination, or stretching of the brainstem producing symptoms^54-56^. Assessments have included using flexion and extension in an upright MRI, although the measurements can be made on a standard supine c-spine flexion MRI to include the Clivo-axial angle (CXA), the Harris, and the Grabb Oakes (pBC2) measurements (Henderson FC et al., 2018; Henderson et al., 2019; Henderson et al., 2020) (Fig. 5). These measurements, the assessment of instability rather than hypermobility, and the optimal treatment remain controversial (Brodbelt and Flint, 2017).

Instability has been suggested to be the cause of the symptoms in CM-I (Goel, 2015a). The initial study was based on a group of patients with a very high level of basiler invagination and craniocervical abnormalitites in a centre taking referrals, often after initial CM-I decompressive surgery, from a large population base (Goel, 2015b). Other authors have not found this to be representative of the CM-I population at large. These studies and those related to patients with hypermobility, do suggest that hypermobility or instability at the craniocervical junction may be present in some patients and should be evaluated both clinically and radiologically.

There has been interest in the use of upright MRI in CM-I, as some patients are only symptomatic when erect, and dynamic views can be easier to acquire (Botchu et al., 2018). The tonsils may be lower when upright, and in severely orthostatic symptomatic patients, this may help evaluate surgical options.

### 2.10 Magnetic resonance imaging (MRI) – spinal evaluation

#### 2.10.1 Syringomyelia

Up to 50% of patients with a symptomatic CM-I will develop a syrinx (Holly and Batzdorf, 2019) (Fig. 3). Syrinxes are best shown on sagittal MR T2-weighted imaging of the whole spinal cord, accompanied by axial T2 views. Contrast-enhancing sequences in patients with a syrinx in the absence of a CM-I are mandatory to look for an associated tumour, but are less useful when a CM-I is present (Timpone and Patel, 2015). Syrinx formation is more common in patients with greater tonsillar herniation, and CSF flow obstruction, and are most common at the C4 to C6 levels (Strahle et al., 2011; Strahle et al., 2015). A terminal syrinx, located in the distal spinal cord, is often associated with a tethered cord, or spinal dysraphism. Preoperative radiological identification of a syrinx, even in the absence of associated symptoms, aids surgical decision making and post operative assessment of success.

#### 2.10.2 Tethered cord syndrome

Tethered cord syndrome (TCS) occurs in 14% of patients with CM-I (Milhorat et al., 2009). Whilst the term tethered cord can indicate a fixed area of spinal cord, TCS refers to tethering of the spinal cord at the lumber level (Hertzler et al., 2010). This is usually diagnosed when the conus medularis is caudal to L2, but other radiological signs used include a thickened or fatty filum, spina bifida oculta, terminal syringomyelia, a lower thoracic scoliosis, and a dorsal position of the filum on prone or upright MRI (McGirt et al., 2006). Lumbar MRI demonstrates the level of the conus medullaris, the thickness of the filum terminale, and any associated dysraphic elements. Further assessment with CT for more complex bony abnormalities, and electrophysiology for urological impairment, is sometimes required. Whilst release of a ‘classical’ radiologically tethered cord in a patient with a symptomatic CM-I and/or terminal syrinx is accepted treatment, some authors will divide a normal filum in treating patients with CM-I, but this remains controversial (Seki et al., 2016; Tandon et al., 2014).

## 3 Conclusions

The majority of CM-I diagnoses are incidental findings but can lead to great anxiety on the part of the non specialist physician and patient. A MRI brain and cervical spine is indicated in all such radiologically labelled CM-1. The radiology report should attempt to comment on any underlying cause for CM-1, the degree of tonsillar descent, impaction and obliteration of CSF spaces. All such cases should be discussed with the appropriate specialist and clinically assessed as appropriate by a detailed history and examination. In radiologically significant CM-I cases and/or symptomatic patients, MRI brain and entire spine is indicated. Radiological significance is denoted by the presence of significant tonsillar impaction in CM-I resulting in tonsillar asymmetry or loss of CSF spaces around the tonsillar region, or other associated structural abnormalities. Dynamic views of fluid flow, and flexion/extension MRI and localised detailed CT scanning can also aid in surgical decision making and help predict surgical success. Development of a grading scheme based on specific MRI characteristics may help identify patients likely to develop symptoms and predict surgical outcomes.

## Presentation at a conference

Syringomyelia-Chiari 2018 International Symposium.

## Clinical trial registration number

Not required.

## Funding

No funding was received for this research.

## Conflict of interest

All authors certify that they have no affiliations with or involvement in any organization or entity with any financial interest (such as honoraria; educational grants; participation in speakers' bureaus; membership, employment, consultancies, stock ownership, or other equity interest; and expert testimony or patent-licensing arrangements), or non-financial interest (such as personal or professional relationships, affiliations, knowledge or beliefs) in the subject matter or materials discussed in this manuscript.

## Ethical approval

This article does not contain any studies with human participants performed by any of the authors.

## Declaration of competing interest

The authors declare that they have no known competing financial interests or personal relationships that could have appeared to influence the work reported in this paper.
